# Data the DEHP induced changes on the trace element and mineral levels in the brain and testis tissues of rats

**DOI:** 10.1016/j.dib.2019.104526

**Published:** 2019-09-17

**Authors:** Duygu Aydemir, Gözde Karabulut, Muslum Gok, Nurhayat Barlas, Nuriye Nuray Ulusu

**Affiliations:** a Koc University, School of Medicine, Department of Medical Biochemistry, Sariyer, 34450, Istanbul, Turkey; b Koç University Research Center for Translational Medicine (KUTTAM), Sariyer, 34450, Istanbul, Turkey; c Dumlupınar University, Faculty of Science, Department of Biology, Kütahya, Turkey; d Hacettepe University, Faculty of Medicine, Department of Medical Biochemistry, Ankara, Turkey; e Hacettepe University, Faculty of Science, Department of Biology, Ankara, Turkey

**Keywords:** Di (2-ethylhexyl) phthalate, Blood-brain barrier, Testis-blood barrier, Antioxidant enzymes, Trace element and minerals

## Abstract

Di (2-ethylhexyl) phthalate (DEHP) is used as plasticizer in the industry and belongs to the phthalate family which can induce tissue damage including kidney, liver, and testis as a result of elevated oxidative stress levels. Glutathione reductase (GR), Glucose-6-phosphate dehydrogenase (G6PD), glutathione S-transferase (GST), 6-phosphogluconate dehydrogenase (6PGD), enzyme activities, trace element and mineral levels were evaluated in the brain and testis tissue samples. Our data revealed that, antioxidant enzyme activities in the brain and testis samples were statistically insignificant in the DEHP administered groups compared to the control group except 400 mg/kg/day DEHP dose group in the testis samples. DEHP can disrupt trace element and mineral levels unlike antioxidant enzyme levels that may due to blood-brain and testis-blood barrier and/or short-term exposure to the DEHP. For more detailed information than the data presented in this article, please see the research article “Impact of the Di (2-Ethylhexyl) Phthalate Administration on Trace Element and Mineral Levels in Relation of Kidney and Liver Damage in Rats” [1].

**Table:** Specifications Table

Subject	Biology
Specific subject area	Endocrine disrupting chemicals (EDCs)
Type of data	Graphs Tables
How data were acquired	Spectrophotometer, ICP-MS Microwave digestion Animal experiments
Data format	Raw and Analyzed
Experimental factors	Rats were exposed to the DEHP administration (0, 100, 200 and 400 mg/kg/day). Antioxidant enzyme activities, trace element and mineral levels were investigated in the rat brain and testis samples
Description of data collection	Enzyme activities were measured via spectrophotometer. Tissue samples were prepared via microwave digestion. ICP-MS was used to determine trace element and mineral levels in the brain and testis samples of the DEHP administered rats.
Data source location	Medical School of Koc University, Istanbul, Turkey
Data accessibility	All data are provided in this article. Raw data is available as supplementary file.
Related research article	Duygu Aydemir, Gözde KarabulutGülsu Şimşek, Muslum Gok, Nurhayat Barlas, Nuriye Nuray Ulusu Impact of the Di (2-Ethylhexyl) Phthalate Administration on Trace Element and Mineral Levels in Relation of Kidney and Liver Damage in Rats Journal Biological Trace Element Research https://doi.org/10.1007/s12011-018-1331-0 All data used in this article is unpublished

**Table:** 

**Value of the data** • This research evaluates the impact of the DEHP on the antioxidant metabolism and trace element and mineral levels on the rat brain and testis first time in the literature. • ICP-MS data is useful for the information about DEHP-induced changes in the trace element and mineral levels. • These data are relevant in both toxicology and biochemistry research, especially for the understanding of effects on the endocrine disrupting chemicals on the tissues with the blood-tissue barrier. • The data are useful for the revealing anti-oxidant enzyme status and imbalance in the trace element and mineral levels upon DEHP treatment at the different concentrations.

## 1 Data

**Fig. 1:**
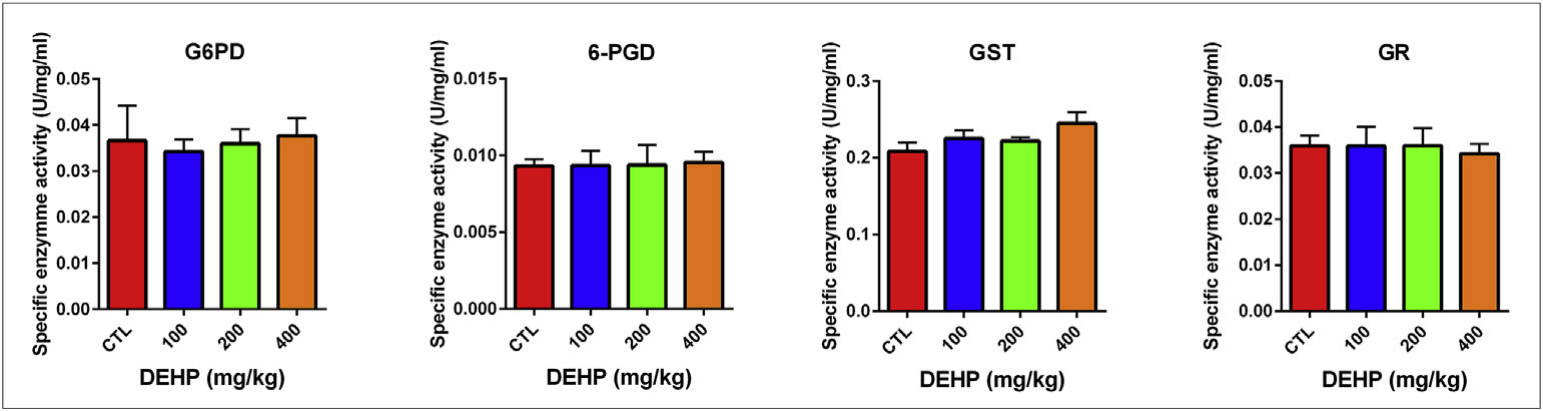
Activities of G6PD, 6PGD, GST, GR enzymes in brain of prepubertal male rats in control and treatment groups. All groups are compared to control group and each other. All data were given as the mean ± SD of n = 6 animals.

**Fig. 2:**
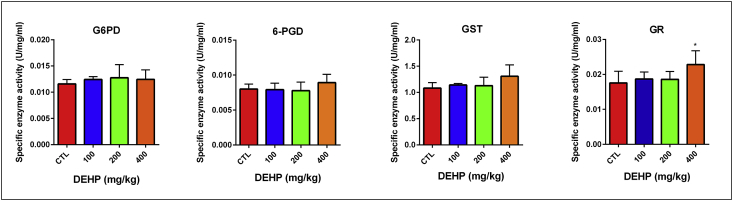
Activities of G6PD, 6PGD, GST, GR enzymes in testis of prepubertal male rats in control and treatment groups. All groups are compared to control group and each other. All data were given as the mean ± SD of n = 6 animals.

**Fig. 3:**
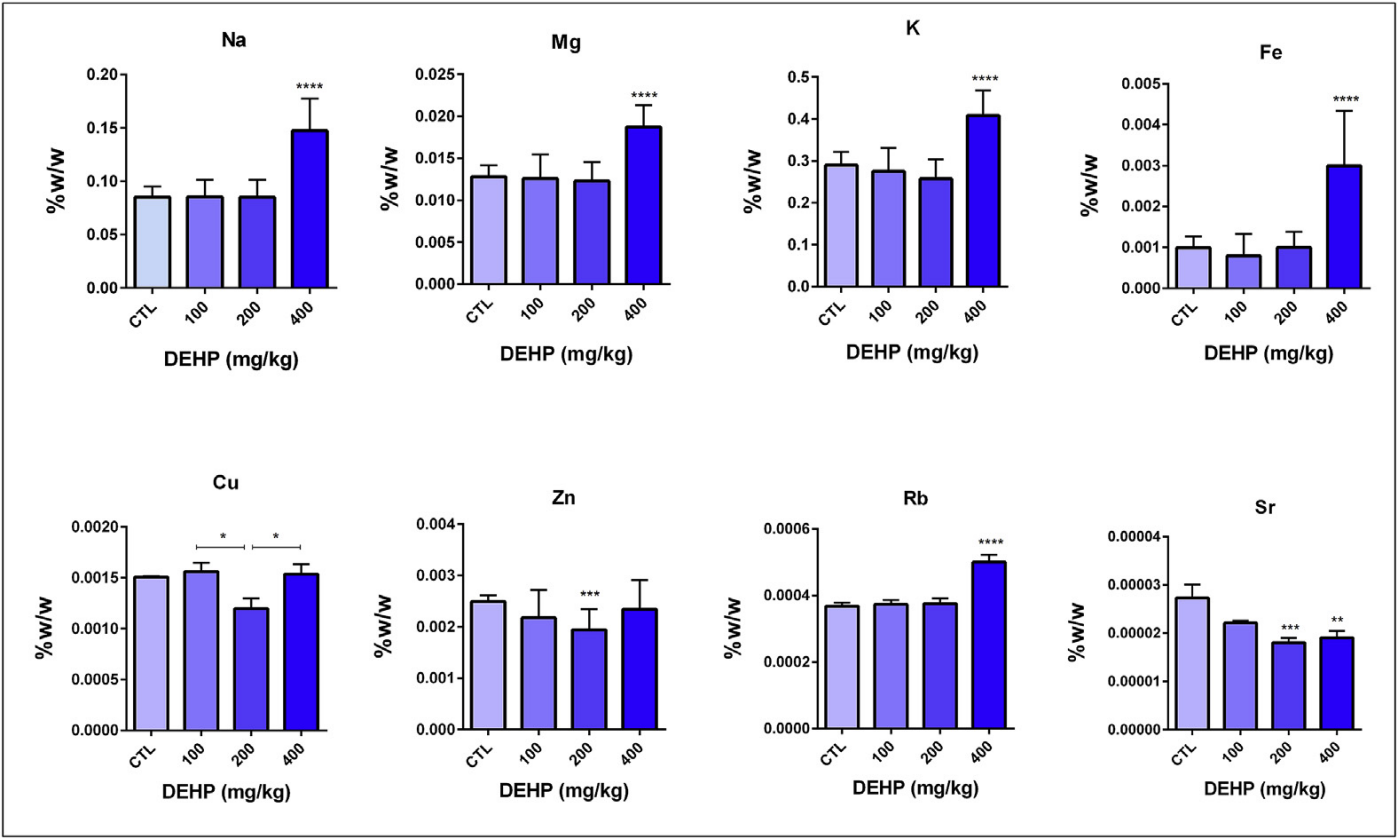
Trace element and mineral levels in prepubertal male rat brain samples. All groups are compared to control group and each other. All data were given as the mean ± SD of n = 6 animals.

**Fig. 4:**
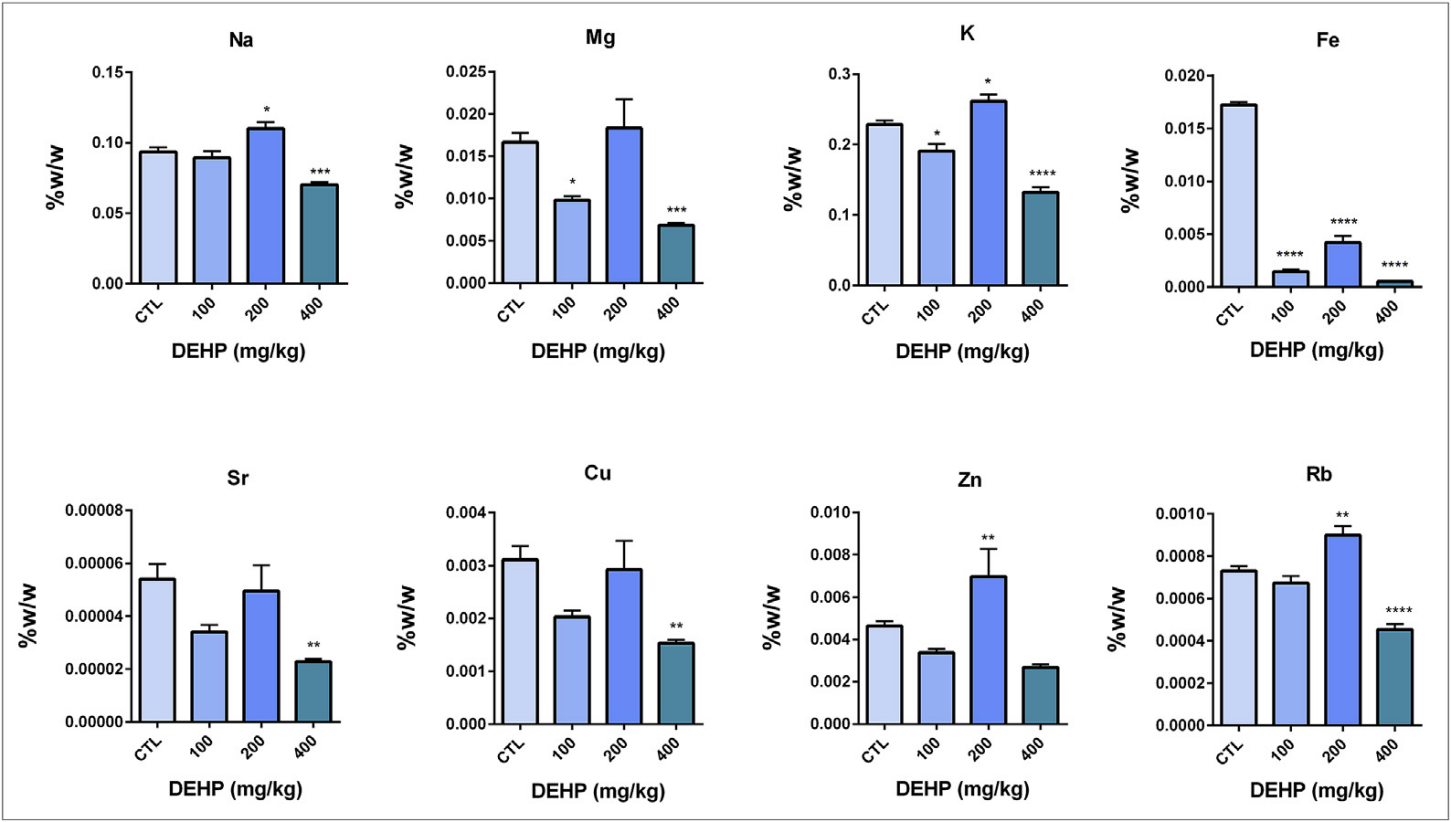
Trace element and mineral levels in prepubertal male rat testis samples. All groups are compared to control group and each other. All data were given as the mean ± SD of n = 6 animals.

DEHP and/or its metabolites can induce organ damage as a result of elevated oxidative stress levels in the different types of tissues [1], [2], [3], [4]. In this report, we showed the mineral and trace element levels and G6PD, GR, 6-PGD and GST antioxidant enzyme activities in the brain and testis samples. Antioxidant enzyme levels did not change in the brain samples unlike testis Fig. 1, Fig. 2, Table 1, Table 2, supplementary data 1). In the testis tissue, GR enzyme activity significantly increased in the 400 mg/kg DEHP treated group compared to the control (Fig. 2, Table 2). In this frame, we can say that the testis is more affected than brain upon DEHP. Sodium (Na), magnesium (Mg), potassium (K), rubidium (Rb) and iron (Fe) levels significantly increased in 400 mg/kg/day DEHP treated groups compared to the control in rat brain tissue samples. However, their concentrations significantly decreased in the 400 mg/kg/day DEHP group compared to the control in the testis samples (Fig. 3, Fig. 4, supplementary data 2).

**Table 1:** Activities of the antioxidant enzymes in the brain of male rats in the control and treatment groups.

Brain	Oil control	100 mg/kg/day DEHP	200 mg/kg/day DEHP	400 mg/kg/day DEHP
Glucose-6-phosphate dehydrogenase (G6PD)	0,0366 ± 0,007	0,0342 ± 0,002	0,0359 ± 0,0013	0,0376 ± 0,0006
6- Phosphogluconate dehydrogenase (6PGD)	0,0093 ± 0,0004	0,0093 ± 0,0009	0,0093 ± 0,0005	0,0095 ± 0,0003
Glutathione-S-transferase (GST)	0,2082 ± 0,0232	0,2251 ± 0,0259	0,2219 ± 0,0112	0,2448 ± 0,0356
Glutathione reductase (GR)	0,0358 ± 0,0023	0,0358 ± 0,0042	0,0359 ± 0,0038	0,03423 ± 0,0021

All results were given as mean ± SD of n = 6 animals.

**Table 2:** Activities of the antioxidant enzymes in the testis of rats in the control and treatment groups.

Testis	Oil control	100 mg/kg/day DEHP	200 mg/kg/day DEHP	400 mg/kg/day DEHP
Glucose-6-phosphate dehydrogenase (G6PD)	0,0115 ± 0,0008	0,0124 ± 0,0005	0,0127 ± 0,0024	0,0124 ± 0,0018
6- Phosphogluconate dehydrogenase (6PGD)	0,0080 ± 0,0007	0,0079 ± 0,0009	0,0077 ± 0,0012	0,0089 ± 0,0011
Glutathione-S-transferase (GST)	1083 ± 0,1038	1140 ± 0,0290	1130 ± 0,1594	1307 ± 0,2184
Glutathione reductase (GR)	0,2082 ± 0,0232	0,2251 ± 0,0259	0,2219 ± 0,0112	0,2448 ± 0,0356^a^

All results were given as mean ± SD of n = 6 animals.

Note: ^a^ p = 0,0343 (P ≤ 0.05).

In conclusion, DEHP induce organ damage in the brain and testis less than kidney and liver that may result from either blood-tissue barriers and/or long-term exposure to the DEHP. In conclusion, DEHP impair mineral and trace element levels in the brain and tissue samples that may cause disease formation in long term exposure.

## 2 Experimental design, materials and methods

### 2.1 Chemicals

Di (2-ethylhexyl) phthalate (DEHP) CAS No. 117-81-7 EC No 204-211-0, glucose-6-phosphate (G6P), reduced nicotinamide adenine dinucleotide phosphate (NADPH + H^+^), 6-phosphogluconate (6-PG), magnesium chloride (MgCl_2_), nicotinamide adenine dinucleotide phosphate (NADP^+^), oxidized glutathione (GSSG), sodium phosphate monobasic and dibasic, Tris (Tris (hydroxymethyl) aminomethane) were purchased from Sigma-Aldrich (USA). 65% nitric acid was obtained from MERCK (Germany).

### 2.2 Animal housing

6 weeks old 24 prepubertal male Wistar albino rats (*Rattus norvecigus*) were obtained from the Experimental Animals Production Center, Hacettepe University in Ankara, Turkey. Our project was approved by the research and ethical committee of the University of Hacettepe 2012/55-03.

### 2.3 DEHP administration

DEHP and corn oil as carrier were administered to the rats for 28 days by daily oral gavage. Rats were randomly divided into the four groups based on the DEHP dosages as 0, 100, 200 and 400 mg/kg/day of DEHP. The dose administration of DEHP was arranged daily basis for the body weight of rats. After 28 days, blood samples were taken from heart after fasting 12 h under ether anesthesia. Animals were sacrificed after decapitation and tissues were collected to store – 80 °C.

### 2.4 Microwave digestion of tissue samples

Microwave digestion system (Milestone START D) was used to prepare brain and testis tissue samples for ICP-MS. 40–80 mg of tissue samples were dissolved in the 10 ml of 65% nitric acid (HNO_3_). First digestion was performed at 150 °C for 15 min and the second digestion at 150 °C for 30 min. Samples were stored at − 20 °C until ICP-MS analysis.

### 2.5 Measurement of the mineral and trace element levels via inductively coupled plasma mass spectrometry (ICP-MS)

Trace elements and minerals of microwave digested tissue samples were measured by the Agilent 7700x ICP-MS (Agilent Technologies Inc., Tokyo, Japan) in rat brain and testis samples. Spex Certiprep Multi-element calibration standard (2A) was used to prepare external calibration solution. MassHunter software was used to analyze the data.

### 2.6 Sample preparation

Brain and tissue samples were washed out from blood with ice-cold sterile physiological saline solution and afterwards samples were prepared as described by Aydemir et al. [4].

### 2.7 Evaluation of the protein concentration

Protein concentration of the samples was measured by the Bradford method by using Spectramax M2 microplate reader in the 96 well plates [5].

### 2.8 G6PD activity

G6PD enzyme activity was measured via LKB Ultraspec Plus (4054 UV/visible; Cambridge, UK) spectrophotometer. Reactin mixture was prepared with 0.6 mM G6P, 10 mM MgCl_2_, 0.2 mM NADP+ in 100 mM Tris/HCl buffer (pH 8.0) and tissue homogenate was used as the enzyme source. NADPH production was determined at 340 nm and 37 °C for 60 sec [6]. One unit (U) of activity was showed as the amount of enzyme required to reduce one mmol NADP^+^/min.

### 2.9 6-PGD activity

6-PGD activity was evaluated using 0.6 mM 6-PG as substrate instead of Glucose-6-phosphate (G6P) in the assay mixture given above for the G6PD activity measurement [7].

### 2.10 GR activity

GR activity was determined by the modified Staal method [8]. The incubation mixture was prepared with 0.2 mM NADPH, 1 mM GSSG and the tissue homogenate in the 100 mM sodium phosphate buffer (pH 7.4). Decrease of the NADPH absorbance at 340 nm was observed at 37 °C for 60 sec.

### 2.11 GST enzyme activity

GST activity was evaluated by measuring the conjugation of GSH with 1-chloro-2, 4-dinitrobenzene (CDNB) as reported by Habig et al. [9]. Reaction mixture was prepared with 200 mM sodium phosphate buffer (pH 6.5), 20 mM CDNB, 20 mM GSH and tissue lysate was used as enzyme source.

### 2.12 Statistical analysis

Statistical analysis was evaluated by the GraphPad Software. One-way analysis of variance (ANOVA) with a Tukey's post hoc test for multiple comparison was performed to analyze each data. All data were showed as the mean ± standard deviation (SD).
